# Draft Genome Sequence of *Enterobacter* sp. Sa187, an Endophytic Bacterium Isolated from the Desert Plant *Indigofera argentea*

**DOI:** 10.1128/genomeA.01638-16

**Published:** 2017-02-16

**Authors:** Feras F. Lafi, Intikhab Alam, Rene Geurts, Ton Bisseling, Vladimir B. Bajic, Heribert Hirt, Maged M. Saad

**Affiliations:** a King Abdullah University of Science and Technology (KAUST), Biological and Environmental Sciences and Engineering Division (BESE), Thuwal, Kingdom of Saudi Arabia; b Computational Bioscience Research Center (CBRC), King Abdullah University of Science and Technology (KAUST), Thuwal, Kingdom of Saudi Arabia; c Department of Plant Sciences, Laboratory of Molecular Biology, Wageningen University, Wageningen, Netherlands

## Abstract

*Enterobacter* sp. Sa187 is a plant endophytic bacterium, isolated from root nodules of the desert plant *Indigofera argentea*, collected from the Jizan region of Saudi Arabia. Here, we report the genome sequence of Sa187, highlighting several genes involved in plant growth–promoting activity and environmental adaption.

## GENOME ANNOUNCEMENT

In an effort to explore the microbial diversity of the desert pioneer plants, the Darwin21 project (http://www.darwin21.net) has been established. Under the project, extensive microbial isolation from the roots of different desert plants has been conducted. Preliminary results revealed a large diversity of bacterial species with a potential to promote the growth of *Arabidopsis thaliana* plants under different biotic and abiotic stresses. A selected number of these strains were sequenced and characterized as described previously (1, 2). *Enterobacter* sp. Sa187 is an endophytic bacterium isolated from surface-sterilized root nodules formed on roots of the pioneer plant *Indigofera* *argentea* Burm. f. (*Fabaceae*). Plants were collected from different regions in the Jizan area (16°56.475′N, 42°36.694′E) of Saudi Arabia. Sa187 has been shown to promote plant growth–promoting activities, such as the production of siderophores and indole acetic acid (IAA). Based on the 16S rRNA gene sequence, strain Sa187 is closely related to *E. kobei* CCUG 49023^T^ and *E. aerogenes* strain KCTC 2190 with 99% sequence similarity (3).

The genomic DNA of Sa187 was extracted using the Qiagen DNeasy blood and tissue kit, following the manufacturer’s protocol. The DNA was then sequenced using paired-end Illumina MiSeq, and the library preparation was constructed as described previously (1). Contig assembly was done with SPAdes assembler version 3.6 (4) with a 1-kb contig cutoff size. *De novo* assembly of MiSeq reads for *Enterobacter* sp. Sa187 resulted in 14 contigs with a total length of 4,404,403 bp and a mean contig size of 314,600 bp. The *N*_50_ was 2,296,004 bp, and the L50 was reached in 1 contig. The G+C content of this draft genome was 56%. MegaBLAST (5) comparison of the Sa187 concatenated contigs against the NCBI reference genome database (http://www.ncbi.nlm.nih.gov/genome) revealed the closest relative genomes being *E. sacchari* SP1 with a coverage of 63% and sequence identity of 95% (accession number NZ_CP007215.2) (6). The annotation of *Enterobacter* sp. Sa187 was carried out using the default INDIGO pipeline (7), with the exception of open reading frames (ORFs) predicted by FragGeneScan (8). The annotation of Sa187 resulted in 3,087 ORFs, 9 rRNAs, 75 tRNAs, and 145 ncRNAs.

The annotation predicted a number of siderophore pathway genes such as *entE*, *entC*, *entA*, *entB*, *entF*, as well as *entS*, an MFS transporter of enterobactin. An ABC transporter involved in iron uptake (*sitABCD*) was also found, as well as five copies of the iron complex outer membrane receptor (*fhu*A), and a TonB-dependent outer membrane iron-enterobactin/colicin (*fepA*). Generally, plant growth–promoting rhizobacteria enhance plant growth through the synthesis of IAA from tryptophan via indole pyruvate as the main pathway (9). The Sa187 genome harbors a number of genes involved in this pathway but lacks the gene encoding for indolepyruvate decarboxylase (*ipdC*). Moreover, the Sa187 genome codes for the enzyme tryptophanase (TnaA) (EC: 4.1.99.1), which can transform tryptophan into indole. Further analysis of the genome sequence of Sa187 will provide valuable genetic information to better understand how the strain interacts with different plants.

### Accession number(s).

The genome of *Enterobacter* sp. Sa187 was deposited at DDBJ/EMBL/GenBank under the accession number MORB00000000. The version described in this paper is the first version, MORB00100000.
